# Digital Representation of Patients as Medical Digital Twins: Data-Centric Viewpoint

**DOI:** 10.2196/53542

**Published:** 2025-01-28

**Authors:** Stanislas Demuth, Jérôme De Sèze, Gilles Edan, Tjalf Ziemssen, Françoise Simon, Pierre-Antoine Gourraud

**Affiliations:** 1 INSERM U1064, CR2TI - Center for Research in Transplantation and Translational Immunology, Nantes University, 30 Bd Jean Monnet, Nantes, 44093, France, 33 2 40 08 74 10; 2 INSERM CIC 1434 Clinical Investigation Center, University Hospital of Strasbourg, Strasbourg, France; 3 Department of Neurology, University Hospital of Strasbourg, Strasbourg, France; 4 Department of Neurology, University Hospital of Rennes, Rennes, France; 5 Center of Clinical Neuroscience, University Hospital Carl Gustav Carus, Dresden, Germany; 6 Department of Health Policy & Management, Columbia University, New York, NY, United States; 7 Mount Sinai School of Medicine, New York, NY, United States; 8 Pôle Hospitalo-Universitaire 11: Santé Publique, Clinique des données, INSERM, CIC 1413, Nantes University Hospital, Nantes, France

**Keywords:** digital twin, artificial intelligence, data architecture, synthetic data, computational modeling, precision medicine, conceptual clarification, conceptual, patient, medicine, health record, digital records, synthetic patient

## Abstract

Precision medicine involves a paradigm shift toward personalized data-driven clinical decisions. The concept of a medical “digital twin” has recently become popular to designate digital representations of patients as a support for a wide range of data science applications. However, the concept is ambiguous when it comes to practical implementations. Here, we propose a medical digital twin framework with a data-centric approach. We argue that a single digital representation of patients cannot support all the data uses of digital twins for technical and regulatory reasons. Instead, we propose a data architecture leveraging three main families of digital representations: (1) multimodal dashboards integrating various raw health records at points of care to assist with perception and documentation, (2) virtual patients, which provide nonsensitive data for collective secondary uses, and (3) individual predictions that support clinical decisions. For a given patient, multiple digital representations may be generated according to the different clinical pathways the patient goes through, each tailored to balance the trade-offs associated with the respective intended uses. Therefore, our proposed framework conceives the medical digital twin as a data architecture leveraging several digital representations of patients along clinical pathways.

## Introduction

**Figure 1.**
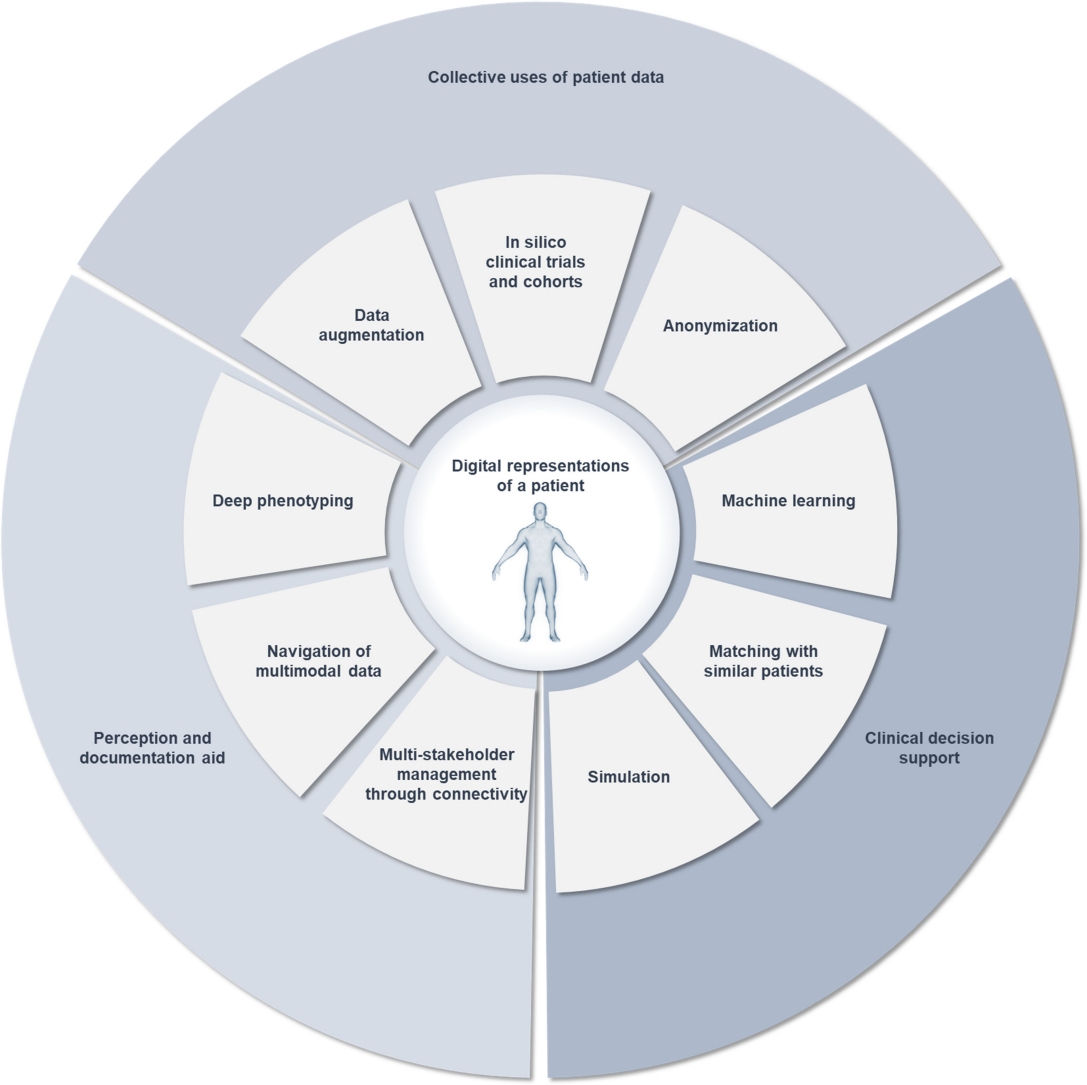
Intended data uses related to digital twins in precision medicine. They rely on various ways of representing patients digitally. Each one may be designated as a “digital twin” in the literature, yielding ambiguity.

Much has been published about digital twins as a landmark of the digital transition of medicine and as a technology to address the uniqueness of patients in a precision medicine framework [1]. The digital twin concept combines engineering technologies attempting to represent objects digitally while maintaining a continuous connection with the physical object in the real world [2]. The manufacturing industry uses digital twins to model physical assets computationally to optimize procedures along their life cycles such as in silico prototyping, production of regulatory evidence, and predictive maintenance [3]. However, in health care, the term “digital twin” may refer to 2 distinct frameworks. One is industrial and aims at representing medical devices digitally and their physiological environments [4]. The other is medical and aims at representing patients digitally in the context of a medical procedure [5]. As such, medical digital twins embody a paradigm shift in the intentionality of health data, from health records to actionable digital representations of patients supporting various data science applications in health (Figure 1).

Representing patients digitally involves 2 major challenges compared to engineering: the complexity of the represented system and the sensitivity of patients’ data. As the term “digital twin” gains popularity, confusion arises from its use to designate a wide range of data usage in health [6], summarized in Figure 1. As mentioned, from an industrial viewpoint, it may designate models of medical devices along their product life cycle [7] or patients in a virtual cohort to run in silico trials [8]. From a management viewpoint, it may designate software agents for care coordination during trauma management [9]. In a patient-centric view, it may designate a multi-stakeholder documentation system, enabling health care providers (HCPs) and patients to visualize multimodal data comprehensively [10]. In data management, it may designate the most similar record to a patient found by a matching algorithm in a reference database [11,12]. In modeling, it may designate a biomechanistic model of a body part, such as circulatory systems [13,14] or digital hearts [15]. Such broad usage of the term led to ambiguity about the nature of a medical digital twin in practice.

**Figure 2.**
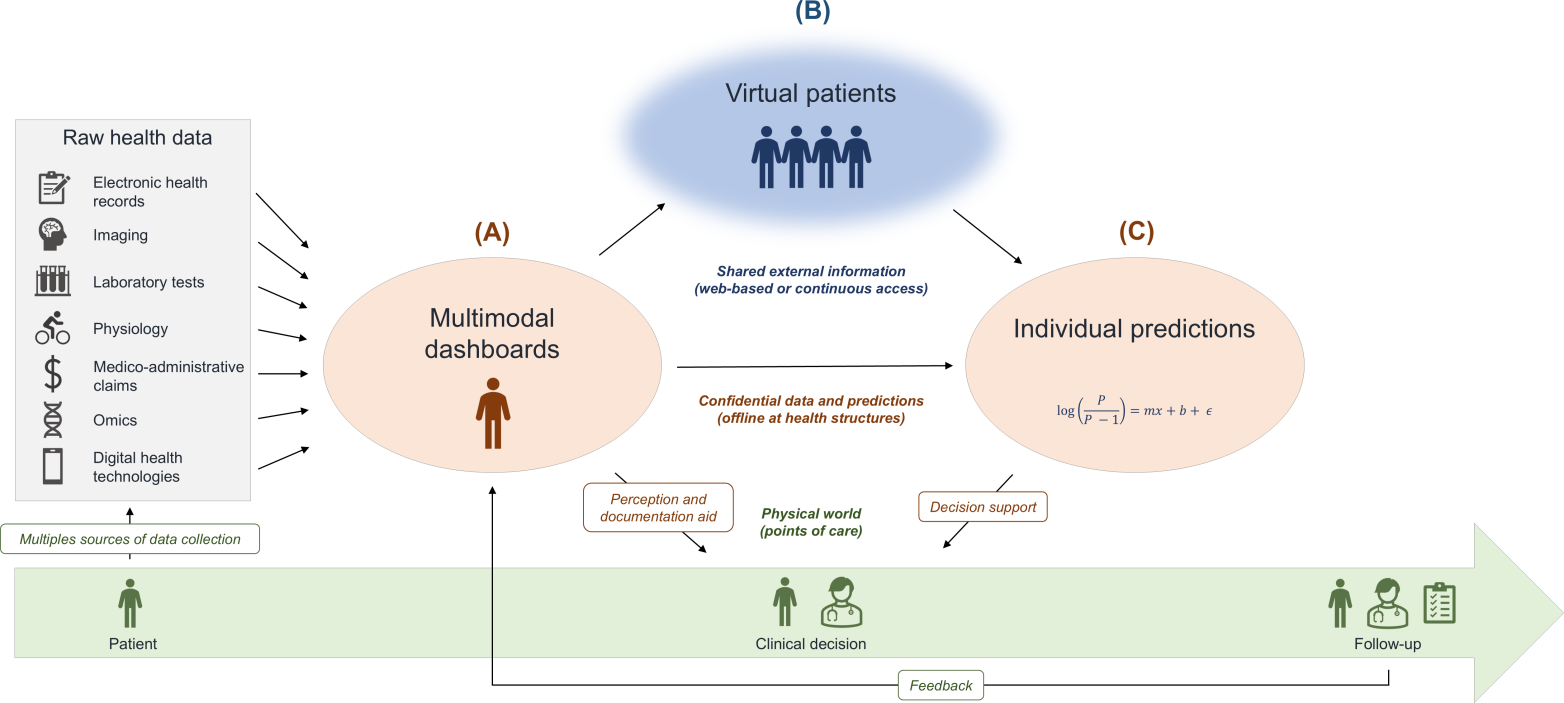
The proposed medical digital twin framework is a data architecture leveraging 3 main families of digital representations of patients. Patients are encountered at points of care (green timeline). Their data are collected in different records from multiple stakeholders (physicians, nurses, patients, etc) and modalities of investigation. These data are raw materials to be refined into different digital representations with different values. (A) The comprehensive visualization of patient data into multimodal dashboards may provide perception and documentation aid. Such data are strictly personal and confidential (orange). (B) Virtual patients may be generated as proxies of sensitive datasets to share their information content as anonymous data (blue). Careful trade-offs about utility and privacy make them useful for collective secondary uses such as the development of predictive analytics. (C) Individual predictions enrich multimodal dashboards with external information provided by predictive analytics. This data architecture also enhances follow-up by structuring the collection of data during the procedures of the corresponding clinical pathways.

Here, we propose a framework for the implementation of medical digital twins from a data-centric perspective. We explain why digital representations of patients are limited due to technical and regulatory constraints. We propose three main families of digital representations (Table 1 and Figure 2) and outline their purposes and limitations: (1) multimodal dashboards integrating raw health records at the points of care to assist with perception and documentation; (2) virtual patients, which provide nonsensitive data for collective secondary uses; and (3) individual predictions that support clinical decisions along clinical pathways and medical procedures. We conclude that a single digital representation of patients cannot support a medical digital twin. Instead, we recommend designing data architectures, leveraging multiple digital representations of the same patient, whose characteristics would be determined by predefined data uses.

**Table 1.** Definition of the 3 types of digital representations.

Digital representation	Definition	Purpose	Limits
Multimodal dashboard	Comprehensive visualization of multimodal data	Perception and documentation aid	Only retrospective Regulatory obstacles to data sharing
Virtual patient	Computer-generated observations	Collective value	On-purpose generation Loss of the connection to the original patient
Individual prediction	Results of predictive analytics and the input preprocessed data	Clinical decision support	Need of data preprocessing Typically instantiated once

## Multimodal Dashboards to Provide Perception and Documentation Aids

### Definition and Purpose

The first family of digital representations is the multimodal dashboard. It fetches the raw health records of a patient at the point of care from various data modalities and disparate sources across an institution’s information system or a national health system. These sources may be electronic health records (EHRs), imaging, laboratory tests, physiological tests, medico-administrative claims, and more recently, telemedicine through digital health technologies. The latter may include wearable medical devices and web platforms to collect patient-reported outcome measures. The comprehensive collection of these modalities of data into multimodal dashboards aims to provide HCPs with actionable visualizations to aid their perception of a patient’s health status and history (Figure 2A).

### Capture of Deep Phenotypes

The state-of-the-art digital representation of patients is a set of documents. Typically, general-purpose EHRs (also called “documentation systems”) only capture medico-administrative, treatments, or diagnosis codes in a structured fashion. The history and phenotypic details of the patients are captured as text data from documents and clinical notes, despite efforts to encode them into interoperable concepts [16,17]. This limits dashboards to medico-administrative timelines. Specialized EHRs encourage comprehensive structured data collection, also known as deep phenotyping [18]. Their dashboards can thus provide individual timelines relevant to particular diseases, showing trajectories of specialized concepts (eg, a disability rating scale or a specific biomarker) and histories of disease-modifying treatments. For patients with multiple sclerosis (MS), this is featured by the European Database for Multiple Sclerosis (EDMUS) and Multiple Sclerosis Documentation System 3D (MSDS 3D) [19,20]. The structured data collection about key features of MS diagnosis and follow-up enables them to provide neurologists with summary timelines. The collected data also feed the French and German national MS registries, respectively. Disease registries are currently an invaluable tool for clinical research as they enable retrospective cohort studies to be performed on high-volume databases with specialized concepts that could not be extracted from general-purpose EHRs [21-23]. A limit of the “registry era” is that patients are typically represented only in tabular data. It is a loss of granularity because it requires HCPs to extract the features from raw imaging and signal data. It may also aggregate the information such as representing a clinical phenotype as a 1D disability scale rating. The active data collection yields a problematic amount of missing data, especially during outpatient follow-ups. As such, completion and data quality management by research assistants is usually required.

### Navigation of Raw Multimodal Data

Multimodal dashboards aim at fetching data passively and provide innovative interfaces. The MS BioScreen is an iPad (Apple Inc)-based dashboard illustrating the passive integration of the clinical, biological, and raw imaging data relevant to the assessment of patients with MS [24]. This academic software fetched data collected for the EPIC cohort at the University of California, San Francisco. In addition to the specialized individual timeline, neurologists could navigate the different modalities of raw data (imaging, functional tests, genetics, etc) through the touchscreen to obtain a comprehensive view of the patient’s status. Likewise, the navigation of 3D reconstructions of imaging data (sometimes called “digital clones” [25]) may support surgery planning through augmented reality [26] or robot-assisted procedures [27]. Current limits of secondary data collection include the lack of interoperability at the scale of national health systems [28]. There are efforts to develop “EHR-agnostic” platforms such as BRIDGE (University of California, San Francisco) [29]. It relies on interoperable standards to first fetch data from multiple sources and then provide customizable clinic-specific dashboards implemented as Substitutable Medical Applications and Reusable Technologies (SMART) on Fast Healthcare Interoperability Resources apps. Efficient and reliable data linkage between the various sources is critical to synchronize the records [30].

### Management by Multiple Stakeholders Through Connectivity

Interoperable web-based records promise to enable multiple stakeholders to access dashboards and to contribute to the data collection along with patient follow-up. In a participative approach, patients themselves may visualize their data to support their self-management, as proposed by the open version of the MS BioScreen project [31]. Medical procedures may be continuously monitored by collecting data streams, either during a surgery procedure to give real-time feedback to the operator or along so-called “integrative digital clinical pathways” for outpatients [32]. For instance, data integration from multiple devices has been developed as an agent-based care coordination framework along a clinical pathway of severe trauma management [9]. Process-“digital twins” were developed as web microservices collecting data from the prehospital and in-hospital phases and making it accessible to the different stakeholders through their respective software agents in a multi-agent system environment. In a quality management approach, the data collection about medical procedures through MSDS 3D integrates the concept of digital clinical pathways. Its interface generates dynamically a quality matrix according to the patient’s diagnosis and prescriptions [10]. Quality matrices are visual summaries derived from a set of checklists completed by all relevant stakeholders. They later support the optimization of the clinical pathways.

### Summary and Limits

Therefore multimodal dashboards would be the perceptive side of the medical digital twin framework and would support the connectivity between the twin and the patient. One patient’s data could be displayed by multiple dashboards, each one adapted to the role of each HCP in the relevant clinical pathway. The first limit would be their retrospective nature as external information is needed to give prospective insights and support decisions [33]. The second limit is the regulatory obstacles to sharing patient data, especially for other purposes than the care of the respective patients. Concerns about privacy and consent-restricted secondary uses of patient data have led to regulatory frameworks such as the general data protection regulation in Europe [34]. Patient data are personal, sensitive, and parsimoniously collected from and for the patient. Their collection remains centered on their primary use (ie, personal care), disregarding other secondary uses in research or clinical decision support for other patients. As a consequence, the primary personal and secondary collective uses of data are split (Table 2). The first may be done individually and confidentially. The second relies on the transfer of pseudonymized data between health care and research structures. However, medical practice in a medical digital twin framework would require continuous access to external information through reference data and predictive analytics (Table 2). Data sharing is still underdeveloped in health care because of the loss of usage control and because pseudonymized data only prevents direct reidentification [35].

**Table 2.** Key distinctions made by the proposed data architecture.

	Concept 1	Concept 2
Data processing	Data collection (full granularity)	Data preprocessing for a predictive analytic (formatted for specific analytics)
Data exchange and access	Data transfer (restricted exchange between 2 organizations)	Data sharing (continuously accessible data)
Data usage (and values)	Primary (personal care)	Secondary (research and care of others)
Data privacy	Pseudonymized (deidentified)	Anonymized (unlinked to the source patient)
Purpose	Epidemiology (optimize decisions at the populational-level)	Precision medicine (optimize decisions at the individual-level)

## Virtual Patients for Collective Uses of Health Data

### Definition and Purpose

The second family of digital representations is the virtual patient. It is an individual observation in a set of computer-generated observations called “synthetic data” [36]. The generation of synthetic datasets might be arbitrary, random, rule-based, or simulated from statistical or machine-learning models. Synthetic data have recently gained popularity as a technology that could facilitate secondary data uses (Figure 2B) [36,37]. An example of external information supporting medical practices is the use of normative datasets to define reference ranges for quantitative biomarkers or to standardize biomarker values according to a population distribution [38,39]. They may also help interpret qualitative biomarkers such as the pathologic significance of genetic variations. However, precision medicine requires access to data of lower granularity to personalize the assessment of patients. At the scale of a single institution, the MS BioScreen illustrates the personalization of various MS biomarkers’ reference ranges according to subgroups of patients with similar profiles [24]. Reference data with individual granularity are also required to develop data-driven predictive analytics. The utility of synthetic datasets stems from (1) the structural similarity (ie, the same level of granularity), (2) the veracity of the information content (ie, the comparison with real data yields the same aggregated results), and (3) indiscernibility (ie, neither experts nor artificial intelligence can distinguish synthetic data from original data).

### Potential to Develop Predictive Analytics Through Data Augmentation

Data-driven predictive analytics are developed through machine learning. In cases where high-volume datasets are not available, synthetic data may augment datasets as a workaround for laborious data collection and expert-demanding data labeling [40]. Data augmentation increases the amount of training data either by generating additional slightly modified data points [41] or by using generative artificial intelligence models, such as generative adversarial networks, variational autoencoders, or large language models [42]. For instance, synthetic magnetic resonance imaging (MRI) images with pathologic features of Alzheimer disease may be generated with a variational autoencoder, yielding increased predictive performances of an analytic predicting the diagnosis from the MRI images [43]. The augmentation may also be restricted to data from specific prediction classes to mitigate class imbalance. Synthetic Minority Over-Sampling Technique (SMOTE) is a common technique to do so [44]. In these cases, the utility of synthetic data comes from the gain of predictive performance on an external validation dataset [40,45].

### Potential to Produce Evidence Through In Silico Clinical Trials and Cohorts

Synthetic data may accelerate the production of scientific or regulatory evidence through in silico studies, which rely on fully synthetic study populations. The VICTRE (Virtual Imaging Clinical Trials for Regulatory Evaluation) trial illustrates the case when real datasets would be too expensive to create [46]. This in silico cross-sectional study compared the performances of a computational reader to detect breast cancer on 31,055 synthetic full-field mammography versus 27,960 synthetic breast tomosynthesis images. Synthetic cohorts may also be generated with longer follow-ups than what could be available in real datasets. In MS, a study generated a longitudinal synthetic cohort with a discrete event simulation model of MS activity and forecast its evolution with a lifetime horizon, although the treatment of interest (ofatumumab) had only been approved in 2021 [47]. The goal was to simulate its prescription as a first-line therapy against a second-line therapy with various delays. The simulation predicted better long-term benefits of ofatumumab when prescribed as a first-line therapy. Such a synthetic dataset makes statistical inference more interpretable. Instead of analyzing the “black box” of the model itself, it uses the model in a generative fashion to represent the information it captured as a cohort of virtual patients, which can be analyzed classically.

### Potential to Share Information Through Anonymization

**Figure 3.**
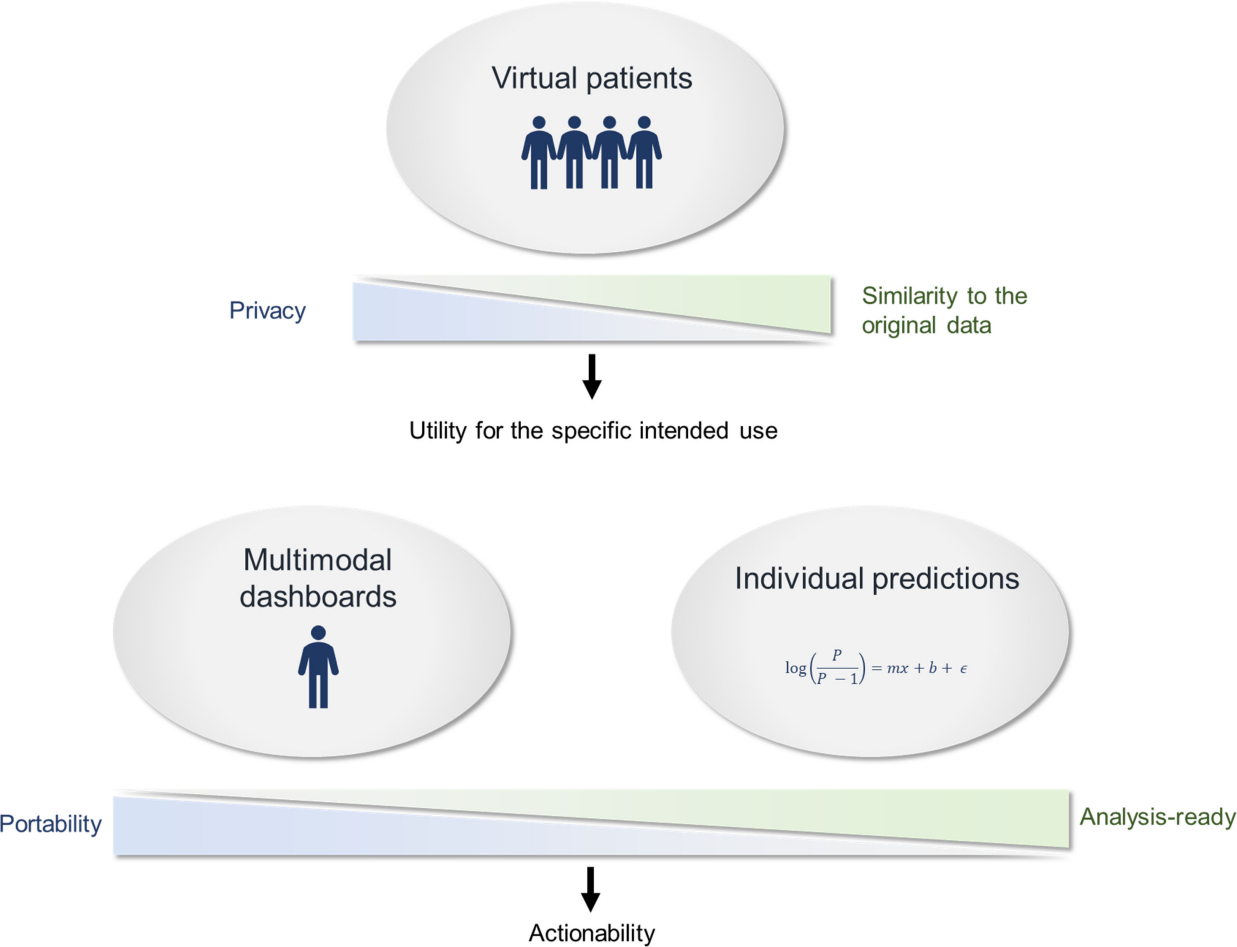
The main trade-offs are addressed by the 3 families of digital representation. Real sensitive patient data may be formatted according to interoperable data models to favor data portability for multimodal dashboards or preprocessed for a specific predictive analytic. Virtual patients must balance privacy and similarity to the original data to be valuable proxies of real identifying records.

Since synthetic data are computer-generated, they are not linkable to a person and are thus assumed to be truly anonymous, as opposed to pseudonymized data. Anonymous data are shareable outside the constraints of regulatory frameworks applied to potentially identifying data. However, synthetic data generators typically do not take privacy protection into account. The generative model is indeed a link between the synthetic and the real data as the information content of the sensitive dataset is represented as a new set of individual observations. Concerns are rising about the risk of linkage between a virtual patient and a real patient (ie, membership inference attacks) [48-50]. Some synthetic data generators are first designed as anonymization techniques, such as the avatars [51]. The avatars take real data as input to generate virtual patients with a probabilistic local model based on the nearest neighbors. The novelty of the method is that it provides privacy metrics to assess that the avatars are no longer identifiable records, even in the case of distance-based membership inference attacks. This privacy-by-design approach to synthetic data generation brings a trade-off [52]. The generation must be destructive enough to protect patients from reidentification while keeping utility for the specific intended use (Figure 3) [37].

### Summary and Limits

In our medical digital twin framework, we argue that virtual patients could be proxies of sensitive patient data to create collective value by sharing the information contained in sensitive datasets. The generation of synthetic data has to be on purpose to prioritize the variables to be represented in the virtual patients and to set the right trade-off between similarity to the original data and privacy. Specific utility for a given use would imply different generation settings (Table 2 and Figure 3). It could be (1) closed-loop software benchmarking with low-similarity synthetic data, (2) medical education [53] or addressing population-level questions in epidemiology (eg, performing a retrospective cohort study) with an intermediate similarity, or (3) supporting individual decisions in precision medicine with high-similarity synthetic data. Therefore, multiple virtual patients could be generated from the data of a given patient. Yet, the connection to the original patient that would be expected from a medical digital twin would be destroyed in all of them. This prevents virtual patients from supporting personal health care and to support individual predictions.

## Individual Predictions to Support Clinical Decisions

### Definition and Purpose

The third family of digital representations is individual prediction. Individual predictions are the results of predictive analytics that may use knowledge-based, data visualization, or model-based algorithms [54]. They enable HCPs to assess alternative scenarios to support clinical decisions such as treatment selection, risk factor prevention, or surgery planning (Figure 2C). The individual predictions of most prognosis scores in medicine use population models [55,56], meaning that decisions are optimized on average at the scale of a population. Medical digital twins aim to optimize decisions at the individual level with personalized analyses.

### Personalization of the Context of Usage of Predictive Analytics

Prognosis scores and predictive analytics in general are validated in restricted contexts of usage [57]. In a chronic disease such as MS, these contexts may be diagnosis, initial prognosis, treatment selection, assessment of therapeutic response, or assessment of the transition to a secondary progressive phase [54]. Therefore management of 1 patient would require the integration of multiple predictive analytics to support different clinical decisions at different points of care along its clinical pathway. Such an approach is conveyed by the digital twin quality management framework of the MSDS 3D [10]. The structuration of health care into digital clinical pathways eases the description of the tree structure of clinical contexts along the management of chronic diseases. As such, different treatment-specific prognosis scores could be used to assess the probability of a patient to respond to each option. This could be further personalized according to the stage or subtype of the disease.

### Personalization of the Analytics to Run Mechanistic Simulations

The most personalized analytics are those modeling the individual. This is the case of organ-level biomechanistic models, such as digital hearts. Their first layer is classically an anatomical mesh of an organ extracted from raw imaging data. The coupling of other modeling layers such as cell and tissue behavior yields an “embodiment” of a generic organ model in each patient’s specific anatomy [5]. Atrial fibrillation (AF) is a case where such modeling is in an early stage of clinical translation [58]. The Optimal Target Identification via the Modeling of Arrhythmogenesis procedure involves a computational model of the left atrium [58,59]. Geometric, fiber orientation, and electrophysiological tissue property layers are simulated to detect the topography of AF re-entrant drivers, including latent ones that electrocardiographic imaging would not detect. It also predicts de novo re-entrant drivers that may later perpetuate arrhythmia according to alternative scenarios of virtual ablation patterns. Thus, the Optimal Target Identification via the Modeling of Arrhythmogenesis procedure integrates the outputs of these simulations to tailor the intervention plan by performing preventive adjuvant ablations of the predicted re-emergent targets [15]. The close-loop of the disease is approached as a multi-scale system and the model enables to simulate emergence phenomena (eg, reentrant drivers) that would not be measurable, or that would occur under various therapeutic scenarios.

### Personalization of the Data Use Based on the Matching With Similar Patients

In cases of statistical modeling, predictive analytics may be personalized by fitting the model at query time only on similar patients recorded in a reference database. The model decision boundaries would thus be optimized in the subset of similar patients matching the patient. The selection of similar patients may be distance-based according to a patient-similarity metric. This may imply a digital representation of patients as data points in a latent reduced multidimensional space, using linear or nonlinear dimension-reduction algorithms [60,61]. On the other hand, the selection of similar patients may be filter-based. The MS-VISTA (Nantes University) prototype of the Projections in Multiple Sclerosis project illustrates the querying of groups of similar patients in an MS randomized clinical trial dataset and the computation of a personalized prognosis to support treatment selection [62]. As such, these analytics personalize the use of continuously accessible reference data.

### Summary and Limits

Individual predictions in our medical digital twin framework would therefore enrich patient data with external information provided by predictive analytics. Unlike one-size-fits-all population models, the analytic would be personalized according to the patient’s characteristics to yield a patient-specific embodiment of the model. One limit is that each model is typically instantiated once at the point of care corresponding to the respective context of usage. Even organ-level models are typically not maintained in the long run, which would be expected from medical digital twins to remain connected to the patient and support further data collection. Another limit is the need to preprocess patient data to run analytics such as the extraction of an anatomical mesh from a stack of raw images. This may involve feature extraction, feature selection, and feature engineering (eg, longitudinal aggregation, criteria fulfilments, events definition from biomarker trajectories, etc). The preprocessed digital representation of a patient therefore loses portability for other data uses (Figure 3). It may require significant computation costs and be subject to an analytic variability that would make it unfit to support personal care outside the context of usage of the analytic. Therefore, even if the analytics are personalized, a medical digital twin would have to leverage multiple shared predictive analytics, each one informing a limited number of decisions or procedures along a given clinical pathway.

## Recommendations

We covered the main digital-twin applications for precision medicine and argued that they cannot all be supported by a single digital representation of a patient due to technical and regulatory constraints. We believe that the clarification of the different digital representations of patients is a foundation for an effective data strategy leveraging various concepts that are currently commonly confounded under the term “digital twins.” We highlighted three main families of digital representations (Figure 2): (1) multimodal dashboards to assist with perception and documentation; (2) virtual patients to facilitate secondary data uses; and (3) individual predictions supporting clinical decisions. For a given patient, multiple digital representations may be generated according to the different clinical pathways the patient goes through, each tailored to balance the trade-offs associated with the respective intended uses (Figure 3).

Therefore the proposed framework conceptualizes the medical digital twin as a data architecture leveraging a multitude of digital representations. It clarifies several distinctions between the characteristics of data usages (Table 2): between data collection and data preprocessing for a predictive analytic, between data transfer and data sharing, between primary personal and secondary collective usages of health data, between pseudonymized and anonymous data, and between population models and personalized analytics. Raw health records are therefore raw material to be refined into various digital representations of patients to fuel precision medicine. Based on this clarification, we propose 3 strategic recommendations, that would ease data architecture efforts to overcome the limitations of the different families of digital representations (Textbox 1).

Textbox 1. **Three recommendations to address the trade-offs of medical digital twin data architectures.**

- Medical digital twin data architectures should relate several digital representations of patients, instead of a single all-encompassing representation.
- Intended data uses should be clearly defined to identify the right family of digital representation to use and to set the right trade-off when generating it.
- For collective usages, real sensitive data should be substituted by synthetic data whenever possible.

## Discussion

This paper proposes a data-centric approach to clarify the practical digital representations at play in a medical digital twin framework from the intended data uses. It does not cover ethical, property, and usage control issues. Clarification efforts about medical digital twins have already been made from other perspectives. Ethical clarification has been proposed about the benefits and risks of medical digital twins [1], as well as about the conditions for medical digital twins to take on ethically justifiable forms of representation [63]. The scope of digital twin applications in the whole health care sector has been reviewed and led to advocate a global collaboration between stakeholders [6]. Clarifications of the means and objectives of the development of “supermodels” have also been postulated [64]. Efforts are made to develop the concept of medical digital twins in the form of computational modeling platforms [65]. In cardiology, a model-centric framework has been formulated, seeking the synergy between deductive and inductive reasoning, respectively conveyed by mechanistic and statistical models [5]. In MS, the clarification has been proposed in a quality management framework [10].

To highlight the perspectives of our framework, we propose road maps for 3 fictional medical digital twin projects (Table 3). Taking the management of AF as an example [58], the multimodal dashboard would collect and integrate all health data relevant to the patient within the clinical pathway of AF management. Second, synthetic heart MRIs would be used to benchmark generic AF heart models. Third, the patient-specific embodiment of an AF heart model would be used to plan an AF ablation procedure [59]. In epilepsy, the patient history and phenotype could be navigated through an epilepsy-specific dashboard. Synthetic electroencephalogram signals could help develop a seizure forecast model [66] or fit a virtual brain model [67] to the patient to support the planning of the ablation of the epileptogenic zone [68]. In MS, patients could complete symptom diaries on patient portals between the visits. They would be integrated with their imaging and therapeutic history in an MS-specific, ophthalmologist-specific, or rehabilitation-specific dashboard depending on the point of care [69]. Virtual patients generated with the avatars [51] could enable the development of a statistical model detecting transitional states to secondary progressive MS [70]. The subset of virtual patients matching the patient characteristics and planned therapeutic scenario could also be analyzed to support treatment selection [62].

**Table 3.** Road maps for fictional medical digital twin projects.

Projects	Multimodal dashboards	Virtual patients	Input and output of predictive analytics
AF^a^	Patient-centric dashboard with anticoagulant treatment plan AF-specific dashboard for the cardiologist	Synthetic heart MRI^b^ for medical education and to benchmark mechanistic organ-level models	Virtual heart model for AF ablation planning.
Epilepsy	Patient-centric dashboard with antiseizure and disease-modifying treatment plans Epilepsy-specific dashboard tailored to the epileptic disease for the neurologist	Synthetic electroencephalogram signals generated with the virtual brain to train deep learning models	Seizure prediction model Virtual brain to plan for the ablation of the epileptic zone
MS^c^	Patient-centric dashboard with treatment plans and symptoms diaries Ophtalmologist-specific dashboard tailored to optic neuritis MS-specific dashboard for the neurologist MS-specific dashboard for rehabilitation	Synthetic cohort of tabular individual patient data generated with the avatars to fit statistical models	Statistical model for detection of transitional state to secondary progressive MS Matching with similar patients to support treatment selection

a AF: atrial fibrillation.

b MRI: magnetic resonance imaging.

c MS: multiple sclerosis.

## Conclusion

We propose a medical digital twin framework as a data architecture leveraging several digital representations of patients, instead of a single all-encompassing representation. The generations of digital representations would be determined by the technical and regulatory constraints of the intended data uses as well as their positioning along clinical pathways.

## Abbreviations

AF: atrial fibrillation
EDMUS: European Database for Multiple Sclerosis
EHR: electronic health record
HCP: health care provider
MRI: magnetic resonance imaging
MS: multiple sclerosis
MSDS 3D: Multiple Sclerosis Documentation System 3D
SMART: Substitutable Medical Applications and Reusable Technologies
SMOTE: Synthetic Minority Over-Sampling Technique
VICTRE: Virtual Imaging Clinical Trials for Regulatory Evaluation
